# Hospital Admissions After Early-Onset Neonatal Bacterial Infection Management Guidelines in France

**DOI:** 10.1001/jamanetworkopen.2025.45436

**Published:** 2025-11-26

**Authors:** Léna Paucard, Bérénice Varga, Elsa Kermorvant-Duchemin, Bich-Tram Huynh, Laurence Watier

**Affiliations:** 1 Université Paris-Saclay, Université de Versailles St Quentin-en-Yvelines (UVSQ), Institut National de la Santé et de la Recherche Médicale (INSERM) 1018, Centre de Recherche en Epidémiologie et Santé des Populations (CESP), Anti-Infective Evasion and Pharmacoepidemiology Research Team, Montigny-Le-Bretonneux, France; 2 Epidemiology and Modelling of Antimicrobials Evasion Team, Institut Pasteur, Université Paris-Cité, Paris, France; 3 Service de Néonatologie et Réanimation Néonatale, Assistance Publique– Hôpitaux de Paris (AP-HP), Hôpital Necker-Enfants Malades, Université Paris-Cité, Paris, France

## Abstract

**Importance:**

The national impact of replacing systematic microbiological sampling with clinical surveillance of asymptomatic newborns at risk of early-onset neonatal bacterial infection (EONI) has not been assessed in France. Specifically, it is not known whether the new guidelines could lead to delayed or missed diagnoses, potentially increasing rates of severe or late-onset infections.

**Objective:**

To assess changes in hospitalization rates for neonatal bacterial infections (NBIs) and their associated costs following the introduction of the 2017 EONI management guidelines in France.

**Design, Setting, and Participants:**

This nationwide, retrospective cohort study was conducted from January 1, 2014, to December 31, 2023. The analysis was based on the French National Hospital Discharge Database, which covers the activity of all public and private health care facilities in France. Participants were newborns of at least 34 weeks’ gestational age (GA) hospitalized for an NBI in the first 28 days of life in metropolitan France.

**Exposure:**

The 2017 French EONI management guidelines.

**Main Outcomes and Measures:**

The primary outcomes were monthly incidence rates of NBI hospitalizations per 1000 live births of at least 34 weeks’ GA between 2014 and 2023. Changes in NBI hospitalization rates following the 2017 management guidelines for EONI were assessed using monthly time series of incidence rates and segmented regression models with autocorrelated errors.

**Results:**

A total of 68 107 EONI hospitalizations were recorded (37 316 male newborns [54.59%]; 61 264 newborns [89.95%] of ≥37 weeks’ GA). The expected incidence rate for nonsevere EONI in March 2018 (start of the postrecommendation period) was 9.13 hospitalizations per 1000 live births (95% CI, 8.27 to 9.98 hospitalizations per 1000 live births), which was significantly higher than the observed incidence rate of 6.17 hospitalizations per 1000 live births (95% CI, 5.74 to 6.61 hospitalizations per 1000 live births) at the same time, corresponding to a relative difference of −32.36% (95% CI, −41.54% to −22.18%). A similar trend was observed for EONI without neonatal intensive care unit admission; the expected incidence rate was 9.46 hospitalizations per 1000 live births (95% CI, 8.56 to 10.36 hospitalizations per 1000 live births), which was significantly higher than the observed incidence rate of 6.68 hospitalizations per 1000 live births (95% CI, 6.22 to 7.13 hospitalizations per 1000 live births), resulting in a relative difference of −29.42% (95% CI, −40.16% to −18.69%). No significant differences were found for severe EONI or EONI requiring neonatal intensive care unit admission. The incidence of late-onset NBI remained stable throughout the study period. Between 2014 and 2023, the total cost for all EONI hospitalizations decreased by half, from €45 975 289 to €23 459 606.

**Conclusions and Relevance:**

In this nationwide cohort study, implementation of the 2017 French EONI guidelines was likely associated with improvements in the management of NBI, contributing to a reduction in nonsevere hospitalizations with no change in the rates of severe cases or late-onset infections.

## Key Points

**Question:**

Were the 2017 French guidelines on early-onset neonatal bacterial infections (EONIs), which replaced systematic microbiological sampling with clinical monitoring for at-risk asymptomatic newborns, associated with changes in hospitalization rates for neonatal bacterial infections?

**Findings:**

In this cohort study of 68 107 hospitalizations for EONI, rates of hospitalizations with a nonsevere EONI diagnosis and those without a neonatal intensive care unit admission were significantly lower following the 2017 guidelines. Rates of severe EONI, neonatal intensive care unit admissions, and vertically transmitted late-onset bacterial infections remained unchanged.

**Meaning:**

These findings suggest that the transition to clinical monitoring for at-risk asymptomatic newborns was associated with fewer hospitalizations and no change in severe or late-onset infections.

## Teaser

This cohort study examines changes in hospitalization rates for neonatal bacterial infections and their associated costs following the introduction of the 2017 early-onset neonatal bacterial infection management guidelines in France.

## Introduction

Neonatal bacterial infections (NBIs) occur within the first 28 days of life and are a substantial cause of morbidity and mortality worldwide.^1^ They can progress to severe, potentially life-threatening conditions, such as sepsis and meningitis,^2,3^ which may have serious neurodevelopmental consequences.^4,5,6,7,8,9^

NBIs are typically divided into 2 groups according to the route of bacterial transmission. Infections attributed to vertical transmission from mother to neonate before or during labor typically manifest within the first 72 hours of life and are usually referred to as early-onset neonatal infections (EONIs). In contrast, infections resulting from horizontal transmission due to postnatal environmental exposure occur more frequently after 72 hours of life and are usually termed late-onset neonatal infections.^10^ Although the incidence rates of culture-confirmed EONI are low in high-income countries (range, 0.7-1.1 cases per 1000 live births; rate in France, 0.32 cases per 1000 live births),^11^ these infections represent a preventable cause of neonatal death.

In high-income countries, group B *Streptococcus* is the leading causative agent of EONI in full-term newborns, whereas *Escherichia coli* is most commonly identified in preterm newborns.^12,13,14^ Risk factors for EONI in full-term and late preterm newborns are well defined and include, but are not limited to, maternal fever during labor (as a proxy for chorioamnionitis), spontaneous preterm birth, maternal group B *Streptococcus* colonization or a history of group B *Streptococcus* in previous pregnancies, prolonged rupture of membranes, and administration and timing of intrapartum antibiotic therapy.^15,16^ Clinical signs of EONI are nonspecific and can mimic some disorders of physiologic transition at birth or other differential diagnoses.^2^ In addition, the absence of reliable early biomarkers makes the diagnosis challenging. In clinical practice, suspected EONI is, therefore, common.

In France, previous guidelines for the management of EONI, published in 2002,^17^ recommended central and peripheral microbiological sampling for all newborns with risk factors for EONI, regardless of symptoms, as well as empiric antibiotic treatment for symptomatic cases and for certain asymptomatic cases on the basis of anamnestic, biological, and bacteriological criteria. This led to excessive sampling, with over 54% of newborns undergoing microbiological testing at birth, and more than 25% having at least 1 invasive blood test.^18^ Additionally, it resulted in overexposure to antibiotics, with nearly 1 in 25 newborns receiving antibiotics,^18^ raising concerns about long-term health outcomes, such as childhood wheezing and asthma, gut microbiome disruption,^19,20,21,22^ and the potential for antimicrobial resistance.^23^

In September 2017, the French health agency Haute Autorité de Santé updated EONI management guidelines for newborns of more than 34 weeks’ gestational age (GA). The new recommendations were intended to promote a rational use of antibiotics and reduce medical intervention in newborns. They aimed to reduce antibiotic use by prioritizing risk-based assessment and close clinical surveillance over bacteriological sampling. The 2017 guidelines primarily impacted asymptomatic newborns at risk, who are now monitored in the maternity ward instead of undergoing microbiological testing or receiving antibiotics. Peripheral sampling has been eliminated, and central sampling with empirical therapy is now reserved for newborns who are symptomatic.^24^

The association between the 2017 recommendations and changes in EONI incidence has not yet been evaluated at a national level. We aimed to assess changes in hospitalization rates with a diagnosis of NBI and their associated costs in France following the introduction of the revised guidelines.

## Methods

### Study Population and Selection of Hospital Stay for Neonatal Infection

We conducted a retrospective cohort study from January 1, 2014, to December 31, 2023, using the French National Hospital Discharge Database (Programme de Médicalisation des Systèmes d’Informations [PMSI]), which covers the activity of all public and private health care facilities in France^25^ (eMethods in Supplement 1). The study population included neonates of at least 34 weeks’ GA, hospitalized within the first 28 days of life with a diagnosis of NBI in metropolitan France. Hospital stays of less than 24 hours were excluded, unless they resulted in death. As this was a retrospective study of an anonymized database, ethics committee approval and informed consent were not required, as per French law. The study was conducted using the Institut National de la Santé et de la Recherche Médicale permanent access to the PMSI. This study followed the Strengthening the Reporting of Observational Studies in Epidemiology (STROBE) reporting guidelines.

NBIs were identified using *International Statistical Classification of Diseases and Related Health Problems, Tenth Revision (ICD-10)* codes from the primary diagnosis, related diagnosis, or significant associated diagnoses fields (eTable 1 in Supplement 1). EONIs were defined as NBIs occurring in the first 3 days of life, whereas late-onset NBIs were defined as NBIs occurring between the fourth and 28th day of life. Infections with sepsis or meningitis were considered as severe infections, and other infections as nonsevere. Neonatal intensive care unit (NICU) stays were considered separately and included admissions to high dependency units and intensive care units to reflect what is typically considered as NICU care in other high-income settings^26^ (eTable 2 in Supplement 1). If multiple *ICD-10* codes were found for a given stay, the most severe code was selected. If multiple severe codes were identified, the one listed as primary diagnosis was retained. Codes for unspecified bacterial infections without sepsis or meningitis, or suspected infections, as well as codes for nosocomial infections were not included (eMethods in Supplement 1). Analyses were conducted for all EONIs, according to infection severity (severe vs nonsevere), level of care (NICU admission vs no NICU admission), for severe infections with an identified pathogen, and for all late-onset NBI.

Annual and monthly numbers of live births of at least 34 weeks’ GA in metropolitan France were extracted using the PMSI database and *ICD-10* codes (eTable 3 in Supplement 1). Variables collected included sex, age, gestational age, length of stay, and vital status at discharge.

### Economic Evaluation

Costs were analyzed from the health care payer’s perspective. Hospital costs for EONI stays were calculated using Diagnosis Related Group (Groupe Homogène de Séjour) costs for public hospitals, covering nursing care, treatments, drugs, accommodation, and medical or technical procedures. Total and average annual costs per hospital stay were calculated and expressed as EONI stays with or without NICU admission.

### Statistical Analysis

Descriptive statistics of newborn and hospital stay characteristics were computed yearly. Age at hospitalization was categorized as up to 24 hours or older, and length of hospital stays was categorized as up to 3 days, 4 to 6 days and 7 days or longer. Qualitative variables were expressed as frequencies (percentages), and quantitative variables were summarized as medians with IQRs. Annual NBI incidence rates and 95% CIs were calculated from 2014 to 2023 and were expressed as the number of cases per 1000 live births of at least 34 weeks’ GA.

To study changes in NBI hospitalization rates following the 2017 French EONI guidelines, monthly time series of incidence rates were constructed using segmented regression models with autocorrelated errors.^27,28^ Two periods were defined: preimplementation (January 2014 to February 2017) and postimplementation (March 2018 to December 2023). The implementation period, covering 6 months before and 6 months after the guidelines’ publication (March 2017 to February 2018), was excluded from the analyses. The 6 months preceding publication were excluded as some clinicians may have anticipated the changes and begun adjusting their clinical practice. For each period, regression lines, including a trigonometric function if annual seasonality was observed, were estimated. If parameters were not significant, they were removed from the model. Model validity was assessed using the Ljung-Box test (independence of residuals) and the Kolmogorov normality test (Gaussian distribution of residuals). Mathematical formulation of the model is described in eMethods in Supplement 1.

Absolute and relative differences between the observed monthly incidence rates and those expected, under the assumption of no change since the preimplementation period, were calculated at the start of the postimplementation phase (March 2018). The 95% CIs for the differences were calculated using a multivariate delta method.^29^ Statistical significance was defined as a 95% CI that did not include the null value of zero. More details can be found in eMethods in Supplement 1. Statistical analyses were computed using SAS Enterprise Guide version 9.4 (SAS Institute) and R statistical software version 4.4.2 (R Project for Statistical Computing).

## Results

### Characteristics of Newborns and Hospital Stays for NBI

**Figure 1.**
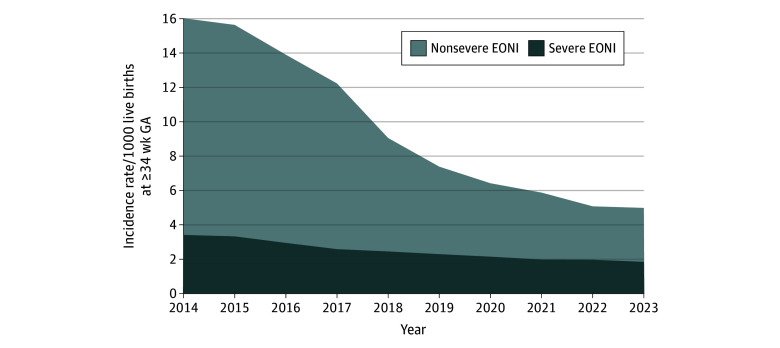
Incidence Rates of Newborns of at Least 34 Weeks’ Gestational Age (GA) Hospitalized for an Early-Onset Neonatal Bacterial Infection (EONI) in France, 2014 to 2023

Over the study period, a total of 68 107 EONI hospitalizations were recorded, including 37 316 male newborns (54.79%) and 61 264 full-term newborns (89.95%). Demographic characteristics such as sex, gestational age, and age at diagnosis remained stable between 2014 and 2023 (Table 1). The number of live births declined from 751 419 in 2014 to 615 840 in 2023, and the number of EONI hospitalizations declined from 12 038 in 2014 to 3069 in 2023. Consequently, the incidence rate of EONI hospitalizations was 16.02 per 1000 live births (95% CI, 15.73 to 16.31 per 1000 live births) in 2014 and 4.98 per 1000 live births (95% CI, 4.81 to 5.16 per 1000 live births) in 2023 (Figure 1). Incidence rates of severe and nonsevere EONI, EONI with and without NICU admission, and late-onset NBI can be found in eTable 4 in Supplement 1. The proportion of hospitalizations lasting 3 days or less increased from 15.49% (1865 hospitalizations) in 2014 to 21.02% (645 hospitalizations) in 2023, whereas stays of 7 days or more increased from 30.98% (3729 hospitalizations) in 2014 to 42.13% (1293 hospitalizations) in 2023. Conversely, hospitalizations lasting 4 to 6 days decreased from 53.53% (6444 hospitalizations) to 36.85% (1131 hospitalizations). The percentage of newborns admitted to the NICU remained steady between 2014 and 2017 but increased notably after 2017, reaching 31.67% (972 newborns) in 2023 compared with 16.63% (2002 newborns) in 2014. Although the crude number of deaths remained stable, the in-hospital mortality rate fluctuated, peaking at 1.52% (51 deaths) in 2022 before declining to 0.94% (29 deaths) in 2023.

**Table 1.** Characteristics of Newborns and Hospital Stays With Early-Onset Neonatal Bacterial Infection per Year in France, 2014-2023

Characteristic	Newborns or hospitalizations, No. (%)										
	Total (N = 68 107)	2014 (n = 12 038)	2015 (n = 11 458)	2016 (n = 9994)	2017 (n = 8589)	2018 (n = 6280)	2019 (n = 5075)	2020 (n = 4310)	2021 (n = 3943)	2022 (n = 3351)	2023 (n = 3069)
Newborn characteristics											
Sex											
Male	37 316 (54.79)	6615 (54.95)	6256 (54.60)	5550 (55.53)	4685 (54.55)	3397 (54.09)	2722 (53.64)	2331 (54.08)	2187 (55.47)	1887 (56.31)	1686 (54.94)
Female	30 791 (45.21)	5423 (45.05)	5202 (45.40)	4444 (44.47)	3904 (45.45)	2883 (45.91)	2353 (46.36)	1979 (45.92)	1756 (44.53)	1464 (43.69)	1383 (45.06)
Age at hospitalization <24 h	64 853 (95.22)	11 548 (95.93)	10 946 (95.53)	9540 (95.46)	8221 (95.72)	5991 (95.40)	4799 (94.56)	4075 (94.55)	3733 (94.67)	3127 (93.32)	2873 (93.61)
Gestational age, wk											
Late preterm (34-36)	6843 (10.05)	1136 (9.44)	1007 (8.79)	957 (9.58)	774 (9.01)	655 (10.43)	479 (9.44)	506 (11.74)	443 (11.24)	488 (14.56)	398 (12.97)
Full term (≥37)	61 264 (89.95)	10 902 (90.56)	10 451 (91.21)	9037 (90.42)	7815 (90.99)	5625 (89.57)	4596 (90.56)	3804 (88.26)	3500 (88.76)	2863 (85.44)	2671 (87.03)
Hospital stay characteristics											
Length of stay, d											
≤3	13 503 (19.83)	1865 (15.49)	2164 (18.89)	1940 (19.41)	1859 (21.64)	1318 (20.99)	1008 (19.86)	1008 (23.39)	1023 (25.94)	673 (20.08)	645 (21.02)
4-6	32 263 (47.37)	6444 (53.53)	5902 (51.51)	5052 (50.55)	4182 (48.69)	2893 (46.07)	2241 (44.16)	1710 (39.68)	1492 (37.84)	1216 (36.29)	1131 (36.85)
≥7	22 341 (32.80)	3729 (30.98)	3392 (29.60)	3002 (30.04)	2548 (29.67)	2069 (32.95)	1826 (35.98)	1592 (36.94)	1428 (36.22)	1462 (43.63)	1293 (42.13)
Median (IQR)	5 (4-7)	5 (4-7)	5 (4-7)	5 (4-7)	5 (4-7)	5 (4-7)	5 (4-8)	5 (4-8)	5 (3-8)	6 (4-9)	6 (4-8)
Neonatal intensive care unit admission	14 212 (20.87)	2002 (16.63)	1897 (16.56)	1736 (17.37)	1523 (17.73)	1328 (21.15)	1323 (26.07)	1181 (27.40)	1113 (28.23)	1137 (33.93)	972 (31.67)
Death	409 (0.60)	43 (0.36)	43 (0.38)	54 (0.54)	39 (0.45)	48 (0.76)	42 (0.83)	29 (0.67)	31 (0.79)	51 (1.52)	29 (0.94)
Gestational age at death, wk											
Late preterm (34-36)	115 (28.12)	9 (20.93)	15 (34.88)	21 (38.89)	9 (23.08)	7 (14.58)	10 (23.81)	13 (44.83)	10 (32.26)	15 (29.41)	6 (20.69)
Full term (≥37)	294 (71.88)	34 (79.07)	28 (65.12)	33 (61.11)	30 (76.92)	41 (85.42)	32 (76.19)	16 (55.17)	21 (67.74)	36 (70.59)	23 (79.31)

### Time Series Analysis

**Figure 2.**
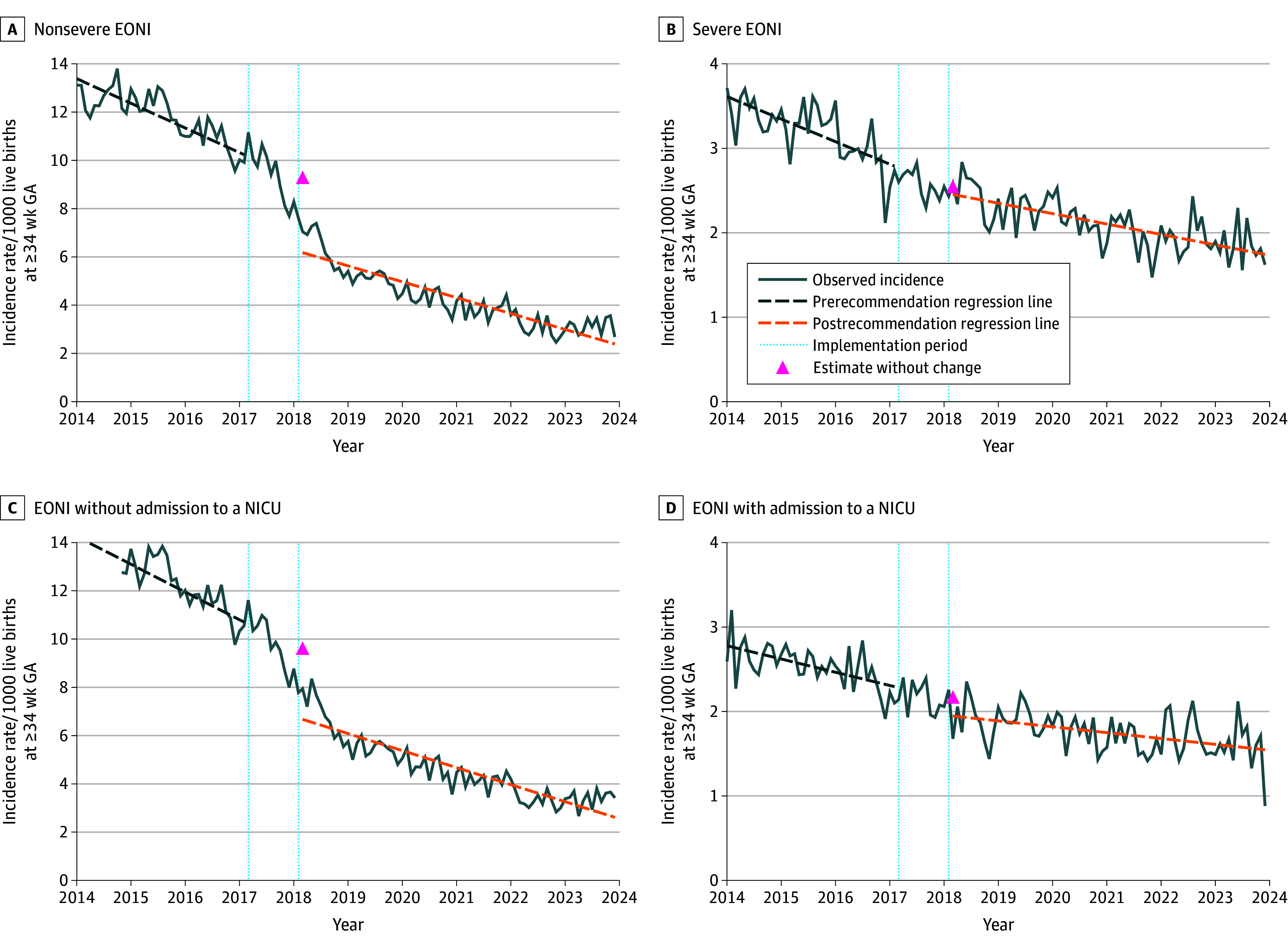
Time Series Analysis of Incidence Rates of Newborns of at Least 34 Weeks’ Gestational Age (GA) Hospitalized for an Early-Onset Neonatal Bacterial Infection (EONI) in France, 2014 to 2023 Graphs show data for nonsevere EONI (A), severe EONI (B), EONI without admission to a neonatal intensive care unit (NICU) (C), and EONI with admission to a NICU (D).

A segmented regression model with autocorrelated error meeting goodness-of-fit criteria was obtained for each series (eTable 5 in Supplement 1). Seasonality was observed in all series before the implementation period, except for EONI with NICU admission. During the preimplementation period, a significant decrease in the incidence rates was observed in all series. After the guideline change, the decrease in incidence rates continued, but the slope significantly decreased slightly in all series, except for late-onset NBI, which remained stable throughout the study period (eTable 6 in Supplement 1). A graphical representation of the time series can be found in Figure 2.

Assuming no association of the new guidelines with the outcomes, the expected incidence rate for nonsevere EONI in March 2018 (start of the postrecommendation period) was 9.13 hospitalizations per 1000 live births (95% CI, 8.27 to 9.98 hospitalizations per 1000 live births), which was significantly higher than the observed incidence rate of 6.17 hospitalizations per 1000 live births (95% CI, 5.74 to 6.61 hospitalizations per 1000 live births) at the same time. This corresponded to an absolute difference of −2.95 hospitalizations per 1000 live births (95% CI, −4.25 to −1.66 hospitalizations per 1000 live births) and a relative difference of −32.36% (95% CI, −41.54% to −22.18%). A similar trend was observed for EONI without NICU admission, with an expected incidence rate of 9.46 hospitalizations per 1000 live births (95% CI, 8.56 to 10.36 hospitalizations per 1000 live births), which was significantly higher than the observed incidence rate of 6.68 hospitalizations per 1000 live births (95% CI, 6.22 to 7.13 hospitalizations per 1000 live births), resulting in an absolute difference of −2.78 hospitalizations per 1000 live births (95% CI, −4.15 to −1.41 hospitalizations per 1000 live births) and relative difference of −29.42% (95% CI, −40.16% to −18.69%). The same pattern was seen for overall EONI.

In contrast, the decreases in the incidence rates observed in the prerecommendation period remained similar in the postrecommendation period for severe EONI, severe EONI with an identified pathogen, and EONI with NICU admission. In addition, late-onset NBI incidence rates remained stable between 2014 and 2023. Estimated variations in incidence rates following the 2017 recommendations can be found in Table 2.

**Table 2.** Estimated Variation in Incidence Rates of Hospitalizations for EONI Among Newborns of at Least 34 Weeks’ Gestational Age After Changes in Management Recommendations

Time series	Incidence rate, No. of hospitalizations/1000 live births (95% CI)		Difference	
	Expected	Observed	Absolute, No. (95% CI)	Relative, % (95% CI)
All EONI	11.66 (10.71 to 12.62)	8.62 (8.14 to 9.10)	−3.05 (−4.50 to −1.59)	−26.12 (−35.73 to −16.51)
Nonsevere EONI	9.13 (8.27 to 9.98)	6.17 (5.74 to 6.61)	−2.95 (−4.25 to −1.66)	−32.36 (−41.54 to −22.18)
Severe EONI	2.50 (2.28 to 2.72)	2.45 (2.35 to 2.56)	−0.05 (−0.39 to 0.30)	−1.81 (−15.30 to 11.67)
Severe EONI with identified pathogen	1.79 (1.60 to 1.98)	1.59 (1.50 to 1.68)	−0.20 (−0.48 to 0.09)	−10.94 (−25.50 to 3.61)
EONI without NICU admission	9.46 (8.56 to 10.36)	6.68 (6.22 to 7.13)	−2.78 (−4.15 to −1.41)	−29.42 (−40.16 to −18.69)
EONI with NICU admission	2.12 (1.91 to 2.33)	1.95 (1.84 to 2.05)	−0.18 (−0.50 to 0.15)	−8.37 (−20.53 to 5.79)
All late-onset neonatal bacterial infection	0.03 (0.02 to 0.03)	0.03 (0.02 to 0.03)	−0.001 (−0.007 to 0.004)	−5.04 (−25.01 to 14.94)

Abbreviations: EONI, early-onset neonatal bacterial infection; NICU, neonatal intensive care unit.

### Economic Evaluation

**Figure 3.**
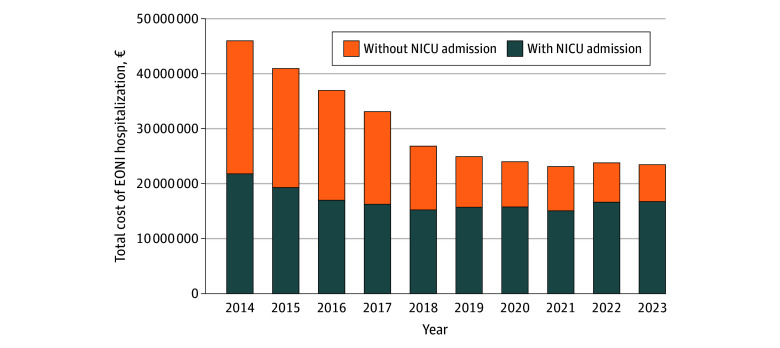
Total Cost in Euros of Hospital Stays for Early-Onset Neonatal Bacterial Infections (EONIs) in France, 2014 to 2023 NICU indicates neonatal intensive care unit.

Between 2014 and 2023, the mean annual cost per stay for all EONI hospitalizations more than doubled, increasing from €4404 (95% CI, €4219-€4590) to €10 305 (95% CI, €9599-€11 011) (eTable 7 in Supplement 1), whereas total costs decreased by half from €45 975 289 to €23 459 606 (Figure 3). Mean costs per stay increased across all care units between 2014 and 2023. Stays with NICU admissions had the highest mean costs and had the most significant increase, from €10 241 (95% CI, €9750-€10 911) to €18 089 (95% CI, €16 748-€19 430), compared with stays without NICU, which increased from €2411 (95% CI, €2373-€2449) to €3197 (95% CI, €3063-€3332). However, total annual costs for stays without NICU admission declined substantially from €24 194 735 to €6 704 929, and total costs for stays with NICU admission total costs showed a slight decrease from €21 780 554 to €16 754 678.

## Discussion

In this nationwide cohort study using the PMSI, we observed a 32.36% relative decrease in hospitalizations for nonsevere EONI and a 29.42% relative decrease in hospitalizations without NICU admission following implementation of the 2017 guidelines. In contrast, the decrease in hospitalizations for severe EONI and EONI requiring NICU admission remained unchanged.

Our findings suggest that the updated guidelines have likely contributed to achieving one of their primary objectives: reducing potentially unnecessary hospitalizations by improving the identification of true cases, consistent with the emphasis on risk-based assessment and clinical monitoring over routine microbiological sampling. Although concerns have been raised that reduced bacterial sampling might delay diagnosis and lead to more severe outcomes, our findings did not support such an association.

Our results are consistent with previous studies suggesting that the introduction of clinical surveillance for at-risk asymptomatic neonates does not lead to more severe outcomes. A study by Schmitt et al^30^ conducted in a single French hospital examining full-term asymptomatic newborns at risk (≥36 weeks’ GA) over a shorter period than our study (2017-2018) found no significant difference in mortality.

Direct comparisons with our study should be made with caution, however, as our cohort included approximately 10% of late preterm newborns between 34 and 36 weeks’ GA, who have a slightly higher risk for severe disease and complications.^31^ The crude number of deaths remained stable; therefore, the increase in in-hospital mortality rates likely reflects the relative increase in the proportion of severe cases, which carry a higher risk of death.

In addition, the stability in late-onset NBI hospitalization rates suggests that clinical surveillance did not lead to missed EONIs subsequently manifesting as late-onset cases. Supporting this finding, a study by Dalut et al^32^ conducted in a single French hospital compared full-term newborns in preimplementation and postimplementation groups and found no statistically significant difference in secondary hospital admission rates among neonates of at least 36 weeks’ GA after clinical monitoring.

After the introduction of the 2017 EONI guidelines, a slight slowdown in the decline of incidence rates was observed. This is consistent with the already low incidence rates achieved. These rates are expected to stabilize at a nonzero level, as indicated by the similar rates observed in 2022 and 2023.

Total costs decreased by half between 2014 and 2023, with a downward trend starting in 2015, prior to the implementation of the guidelines. This reduction was mainly associated with the decrease in stays without NICU admission. Conversely, the average cost per hospital stay increased, parallel to the increase in the proportion of more expensive NICU admissions.

Although the reduction in total hospital costs cannot be directly attributed to the guideline change, it likely reflects fewer hospitalizations for nonsevere infections following implementation, as these cases do not require admission to the NICU. By avoiding unnecessary nonsevere hospitalizations and their related costs, resources can be concentrated on severe cases requiring NICU.

The PMSI database does not collect in-hospital laboratory tests and includes only certain high-cost prescriptions (excluding antibiotics). We hypothesize that the observed reduction of nonsevere EONI hospitalizations may be linked to a decrease in invasive laboratory tests. Indeed, Schmitt et al^30^ reported that the updated guidelines were significantly associated with a reduction in invasive laboratory testing, with the proportion of newborns undergoing at least 1 blood test decreasing from approximately 27% to 1% in the later period. Similarly, a study by Cabaret et al^33^ in a single French hospital found that the implementation of repeated clinical assessments in asymptomatic neonates of at least 35 weeks’ GA significantly reduced the rate of laboratory testing from 100% to 8%.

As antibiotic treatments for EONI are rarely administered outside hospital settings, we assume that antibiotic exposure closely mirrored hospitalization rates. Several studies, both in France and in other countries, have shown reduced antibiotic use with clinical surveillance instead of systematic microbiological sampling.^32,33,34^ For instance, Dalut et al^32^ reported a 50% reduction in antibiotic administration following the guideline change at their center. Similarly, Cantoni et al,^34^ in a multicenter Italian sequential study, compared combined clinical and biological (blood culture and complete blood count) monitoring vs clinical surveillance alone. They found that significantly fewer neonates received antibiotics under clinical monitoring alone (0.5% vs 1.2%; *P* < .001).^34^

### Strengths and Limitations

Our study has several strengths. In contrast to previously published French studies, our study covers a 10-year period and includes all hospitalizations for EONI nationwide, providing a broader scope for analysis. This comprehensive dataset includes all types of NBI cases, including nonsevere cases and meningitis, which are rarely reported in the literature, as most studies primarily focus on sepsis. This allowed an accurate assessment of the new recommendations on all newborns, not just those at risk. To our knowledge, this is the first study to evaluate these guidelines on a national scale, encompassing diverse hospital settings and clinical practices throughout France.

Our study also has limitations. Medical administrative databases provide limited clinical details, which may affect diagnostic accuracy. However, we used widely accepted *ICD-10* codes, and routine audits by the French public health insurance system help minimize coding errors. Moreover, the characteristics of the EONI cases observed in our study align closely with existing literature, with over 95% of cases occurring within the first 24 hours of life.^2^ The lack of bacteriological data may have slightly overestimated true infection rates, as culture-negative cases were included. This broader definition likely accounts for higher rates relative to prior studies, although exclusion of codes for unspecified infections mitigated the risk.

In addition, although our time series analysis reveals notable trends, it does not establish causality. Other factors such as overall antibiotic prescribing patterns, medical advances, and changes in perinatal care may have influenced the results. Furthermore, the implementation of the French guidelines probably varied across health care facilities, potentially attenuating the effects we observed.^30,32,35^ Nevertheless, although countries such as the United Kingdom,^36^ Switzerland,^37^ and New Zealand,^38^ had adopted similar risk-based strategies and clinical surveillance approach prior to 2017, this study provides further evidence of their effectiveness within the known limitations.^39^

## Conclusions

The findings of this nationwide retrospective cohort study support the effectiveness of the 2017 EONI management guidelines, reflected by reduced hospitalization rates for nonsevere infections. This decline in potentially unnecessary hospitalizations contributed to lower EONI-related health care costs. Future research should investigate the association between these revised guidelines and antibiotic exposure in neonates and its related effects on long-term health outcomes.

## Notes

Supplement 1. eMethods eTable 1. *ICD-10* codes used to identify infections with or without sepsis and with meningitis eTable 2. Medical unit codes used to identify hospital stays with neonatal intensive care unit admission eTable 3. *ICD-10* codes used to identify births eTable 4. Incidence rates (per 1000 live births) of newborns of at least 34 weeks’ gestation hospitalized for a neonatal bacterial infection, per year - France, 2014-2023 eTable 5. Estimations of the parameters included in the model for each time series eTable 6. Estimated change in trend in incidence rate per 1 000 live births of newborns of at least 34 weeks’ gestation hospitalized for early-onset neonatal bacterial infection in France due to 2017 guidelines eTable 7. Mean cost in euro per stay of hospitalization for an early-onset neonatal bacterial infection - France, 2014-2023 eReference

Supplement 2. Data Sharing Statement
