# Supplementary material for: Hospital Admissions After Early-Onset Neonatal Bacterial Infection Management Guidelines in France
Source: JAMA Netw Open. 2025 Nov 26;8(11):e2545436. doi: 10.1001/jamanetworkopen.2025.45436 (PMC12658671; doi:10.1001/jamanetworkopen.2025.45436)
Supplement: Supplement 1. — eMethods eTable 1. ICD-10 codes used to identify infections with or without sepsis and with meningitis eTable 2. Medical unit codes used to identify hospital stays with neonatal intensive care unit admission eTable 3. ICD-10 codes used to identify births eTable 4. Incidence rates (per 1000 live births) of newborns of at least 34 weeks’ gestation hospitalized for a neonatal bacterial infection, per year - France, 2014-2023 eTable 5. Estimations of the parameters included in the model for each time series eTable 6. Estimated change in trend in incidence rate per 1 000 live births of newborns of at least 34 weeks’ gestation hospitalized for early-onset neonatal bacterial infection in France due to 2017 guidelines eTable 7. Mean cost in euro per stay of hospitalization for an early-onset neonatal bacterial infection - France, 2014-2023 eReference [file jamanetwopen-e2545436-s001.pdf]

Supplemental Online Content

Paucard L, Varga B, Kermorvant-Duchemin E, Huynh BT, Watier L. Hospital admissions after early-onset neonatal bacterial infection management guidelines in France. *JAMA Netw Open*. 2025;8(11):e2545436.
doi:10.1001/jamanetworkopen.2025.45436

eMethods

eTable 1. *ICD-10* codes used to identify infections with or without sepsis and with meningitis

eTable 2. Medical unit codes used to identify hospital stays with neonatal intensive care unit admission

eTable 3. *ICD-10* codes used to identify births

eTable 4. Incidence rates (per 1000 live births) of newborns of at least 34 weeks' gestation hospitalized for a neonatal bacterial infection, per year - France, 2014-2023

eTable 5. Estimations of the parameters included in the model for each time series

eTable 6. Estimated change in trend in incidence rate per 1 000 live births of newborns of at least 34 weeks' gestation hospitalized for early-onset neonatal bacterial infection in France due to 2017 guidelines

eTable 7. Mean cost in euro per stay of hospitalization for an early-onset neonatal bacterial infection - France, 2014-2023

eReference

This supplemental material has been provided by the authors to give readers additional information about their work.

eMethods

Description of the French National Hospital Discharge Database (PMSI)

Programme de Médicalisation des Systèmes d'Information (PMSI) data includes all discharge summaries of hospitalization and covers all hospital stays in publicly funded and private institutions including acute-care facilities (medicine, surgery or obstetrics units). For each stay the cause for hospitalization is coded using the International Classification of Diseases, 10th Revision (ICD-10) as a unique primary diagnosis (PD: condition requiring hospitalization), a unique related diagnosis (RD: supplementary information to the PD) and as one or more significant associated diagnoses (SAD: complications and co-morbidities that may affect the course or cost of the hospitalization). Additional information is available about the patient, such as sex and age, and about the hospital stays such as admission and discharge date, admission source, hospital discharge or medical procedures.

Identification of neonatal bacterial infection hospital stays

Perinatal neonatal bacterial infections (NBI) were identified using the ICD-10 codes from the PD, RD or SAD fields. ICD-10 codes to identify bacterial meningitis in the perinatal period do not exist, so the code used are those that identify bacterial meningitis in any population. Codes for unspecified bacterial infections without sepsis or meningitis (such as ICD-10 codes P39.99 "Infection specific to the perinatal period, unspecified") or suspected infection (P00.1), as well as codes for nosocomial infections (A40 to A41) were not included.

eTable 1. *ICD-10* codes used to identify infections with or without sepsis and with meningitis

Type of NBI	ICD-10 code
Infection without sepsis or meningitis	P36.09, P36.19, P36.29, P36.39, P36.49, P36.59, P36.89, P37.2
Infection with sepsis	P36.00, P36.10, P36.20, P36.30, P36.40, P36.50, P36.80, P36.90
Infection with meningitis	A32.1, A39.0, G00, G00.0, G00.1, G00.2, G00.3, G00.8, G00.9, G01, G04.2, G05.0

Hospital stays with admission to a neonatal intensive care unit (NICU) were identified using medical unit (MU) codes and included admission to high dependency units and intensive care units.

eTable 2. Medical unit codes used to identify hospital stays with neonatal intensive care unit admission

Label	MU code
High dependency unit	
Neonatal high dependency unit	05
Pediatric high dependency unit excluding major burns	14A
Pediatric high dependency excluding major burns	14B
Intensive care unit	
Neonatal intensive care unit	06
Pediatric intensive care unit excluding major burns	13A
Pediatric intensive care units for major burns	13B

MU: medical unit

Live births in metropolitan France

Annual and monthly numbers of live births in metropolitan France were extracted using the PMSI database and ICD-10 codes. If multiple relevant birth codes were found for a single stay with conflicting information on the number of births, the more conservative code was retained. Births less than six months apart were excluded for mothers with multiple deliveries in the same year.

© 2025 Paucard L et al. *JAMA Network Open*.

eTable 3. *ICD-10* codes used to identify births

Label	ICD-10 code
Singleton birth, live child[a]	Z37.0
Twin birth, twins born alive	Z37.2
Twin birth, one twin born alive, the other stillborn[a]	Z37.3
Twin birth, one twin born alive, the other stillborn, excluding medical abortion[a]	Z37.30
Twin birth, one twin born alive, the other stillborn, following medical abortion [a]	Z37.31
Other multiple births, all born alive[b]	Z37.5
Other multiple births, some live births	Z37.6
Other multiple births, some children born alive, excluding medical abortion	Z37.60
Other multiple births, some children born alive, following medical abortion	Z37.61

[a] These codes count as 1 live birth; [b] These codes count as 3 live births; All other codes not specified count as 2 live births.

Mathematical formulation of the model and estimated changes

Each time series was analyzed using a segmented regression model with autocorrelated errors.

The general formulation of the model can be written as follows:

$$Y_t = \sum_{i=0}^{1} (\beta_{0i} + \beta_{1i}t)\, I_i + \sum_{i=0}^{1} \left(\gamma_i cos\frac{2\pi t}{12} + \delta_i sin\frac{2\pi t}{12}\right) I_i + \nu_t$$

Notation:

t = time index, from 1 to 120,

i = period index, from 0 to 1, i=0 for the pre-recommendation change period (January 2014 to February 2017) and i=1 for the post-recommendation change period (March 2018 to December 2023)

Y_t = number of neonatal infections of interest at time t (month) per 1,000 live births,

I_i = dummy variable for period i,

β_{0i}= intercept parameter for period i,

β_{1i} = slope parameter for period i,

γ_i and δ_i = cosinus and sinus parameters of period i,

ν_t is modelled as an AR(p) process, with residual variance σ^2.

From the model, for each period i (i=0 to 1), the absolute and relative differences in incidence rates between the two periods were assessed at the end of the implementation period. For the post-implementation period (i=1) starting in March 2018 (t*), the estimated level is defined as $\hat{Y}_{t^*} = \hat{\beta}_{01} + \hat{\beta}_{11}t^*$. Assuming no change in the evolution, the predicted level is $\overline{Y}_{t^*} = \beta_{00} + \beta_{10}t^*$.

For each period, the absolute difference (AD) was defined by:

$$AD = \hat{Y}_{t^*} - \overline{Y}_{t^*}$$

And the percentage change (PC) by:

$$PC = \frac{\hat{Y}_{t^*} - \overline{Y}_{t^*}}{\overline{Y}_{t^*}} \times 100$$

If variance of AD can be obtained by using different scripts (e.g. by estimating an intercept over the entire studied period), a delta method approach, as described by Zhang et al. was used to approach the variance of PC[1].

© 2025 Paucard L et al. *JAMA Network Open*.

The variance of $100 \times PC$, which allowed to calculate 95% confidence interval, was defined as follows:

$$Var\left(\frac{\hat{Y}-\overline{Y}}{\overline{Y}}\right) = \left(\frac{\hat{Y}}{\overline{Y}}\right)^2 \left[\frac{Var(\hat{Y})}{\hat{Y}^2} + \frac{Var(\overline{Y})}{\overline{Y}^2} - 2\frac{Cov(\hat{Y},\overline{Y})}{\hat{Y}\overline{Y}}\right].$$

© 2025 Paucard L et al. *JAMA Network Open*.

eTable 4. Incidence rates (per 1000 live births) of newborns of at least 34 weeks' gestation hospitalized for a neonatal bacterial infection, per year - France, 2014-2023

Live births	**2014** N = 751 419	**2015** N = 732 940	**2016** N = 719 105	**2017** N = 702 516	**2018** N = 693 936	**2019** N = 687 352	**2020** N = 671 594	**2021** N = 674 843	**2022** N = 660 331	**2023** N = 615 840
**Total EONI**										
Number of cases	12 038	11 458	9994	8589	6280	5075	4310	3943	3351	3069
Incidence rate	16.02	15.63	13.90	12.23	9.05	7.38	6.42	5.84	5.07	4.98
95% CI	15.73 - 16.31	15.35 - 15.92	13.63 - 14.17	11.97 - 12.48	8.83 - 9.27	7.18 - 7.59	6.23 - 6.61	5.66 - 6.03	4.90 - 5.25	4.81 - 5.16
**Non-severe EONI**										
Number of cases	9474	9019	7875	6773	4580	3499	2868	2626	2053	1933
Incidence rate	12.61	12.31	10.95	9.64	6.60	5.09	4.27	3.89	3.11	3.14
95% CI	12.35 - 12.86	12.05 - 12.56	10.71 - 11.19	9.41 - 9.87	6.41 - 6.79	4.92 - 5.26	4.11 - 4.43	3.74 - 4.04	2.97 - 3.24	3.00 - 3.28
**Severe EONI**										
Number of cases	2564	2439	2119	1816	1700	1576	1442	1317	1298	1136
Incidence rate	3.41	3.33	2.95	2.58	2.45	2.29	2.15	1.98	1.97	1.84
95% CI	3.28 - 3.54	3.20 - 3.46	2.82 - 3.07	2.47 - 2.70	2.33 - 2.57	2.18 - 2.41	2.04 - 2.26	1.88 - 2.09	1.86 - 2.07	1.74 - 1.95
**EONI without NICU**										
Number of cases	10 036	9561	8258	7066	4952	3752	3129	2830	2214	2097
Incidence rate	13.36	13.04	11.48	10.06	7.14	5.46	4.66	4.19	3.35	3.41
95% CI	13.09 - 13.62	12.78 - 13.31	11.24 - 11.73	9.82 - 10.29	6.94 - 7.33	5.28 - 5.63	4.50 - 4.82	4.04 - 4.35	3.21 - 3.49	3.26 - 3.55
**EONI with NICU**										
Number of cases	2002	1897	1736	1523	1328	1323	1181	1113	1137	972
Incidence rate	2.66	2.59	2.41	2.17	1.91	1.92	1.76	1.65	1.72	1.58
95% CI	2.55 - 2.78	2.47 - 2.70	2.30 - 2.53	2.06 - 2.28	1.81 - 2.02	1.82 - 2.03	1.66 - 1.86	1.55 - 1.75	1.62 - 1.82	1.48 - 1.68
**Total late-onset NBI**										
Number of cases	59	54	59	65	67	51	48	40	61	42
Incidence rate	0.03	0.03	0.03	0.03	0.03	0.02	0.02	0.02	0.03	0.02
95% CI	0.02 - 0.04	0.022 - 0.03	0.02 - 0.04	0.02 - 0.04	0.03 - 0.04	0.02 - 0.03	0.02 - 0.03	0.01 - 0.03	0.02 - 0.04	0.02 - 0.03

EONI: early-onset neonatal bacterial infection; NICU: neonatal intensive care unit; NBI: neonatal bacterial infection; 95% CI: 95% confidence intervals

© 2025 Paucard L et al. *JAMA Network Open*.

eTable 5. Estimations of the parameters included in the model for each time series

	EONI total		Non-severe EONI		Severe EONI		Severe EONI with identified pathogen		EONI with NICU		EONI without NICU		Late-onset NBI total	
**Regression parameters** Estimation (standard error), p-value														
β_{00}	17.11 (0.34)	<0.0001	13.45 (0.30)	<0.0001	3.64 (0.08)	<0.0001	2.56 (0.06)	<0.0001	2.79 (0.07)	<0.0001	14.35 (0.32)	<0.0001	0.03 (0.002)	<0.0001
β_{10}	-0.11 (0.01)	<0.0001	-0.09 (0.01)	<0.0001	-0.02 (0.003)	<0.0001	-0.02 (0.003)	<0.0001	-0.01 (0.003)	<0.0001	-0.1 (0.01)	<0.0001	N/A	N/A
β_{01}	11.93 (0.53)	<0.0001	8.97 (0.48)	<0.0001	2.98 (0.12)	<0.0001	1.96 (0.10)	<0.0001	2.24 (0.11)	<0.0001	9.67 (0.51)	<0.0001	0.03 (0.002)	<0.0001
β_{11}	-0.06 (0.01)	<0.0001	-0.06 (0.01)	<0.0001	-0.01 (0.001)	<0.0001	-0.01 (0.001)	<0.0001	-0.01 (0.001)	<0.0001	-0.06 (0.01)	<0.0001	N/A	N/A
**Seasonal parameters** Estimation (standard error), p-value														
γ_0	N/A	N/A	N/A	N/A	N/A	N/A	N/A	N/A	N/A	N/A	N/A	N/A	0.01 (0.003)	0.015
δ_0	-0.55 (0.20)	0.008	-0.44 (0.18)	0.013	0.12 (0.05)	0.032	-0.11 (0.04)	0.014	N/A	N/A	-0.56 (0.12)	0.004	N/A	N/A
**Autoregressive parameters** Estimation (standard error), p-value														
ϕ_1	-0.47 (0.09)	<0.0001	-0.52 (0.09)	<0.0001	N/A	N/A	N/A	N/A	N/A	N/A	-0.51 (0.87)	<0.0001	N/A	N/A
**Residual variance** Estimation														
σ_2	0.33		0.22		0.05		0.04		0.05		0.26		0.0002	
**Diagnostic tests** P-value														
Independence[a]	0.823		0.687		0.730		0.658		0.273		0.645		0.180	
Normality[b]	>0.150		>0.150		>0.150		>0.150		>0.150		>0.150		>0.150	

EONI: early-onset neonatal bacterial infection; NICU: neonatal intensive care unit; NBI: neonatal bacterial infection

[a]Ljung-Box independence of residuals test; [b]Kolmogorov normality test

© 2025 Paucard L et al. *JAMA Network Open*.

eTable 6. Estimated change in trend in incidence rate per 1 000 live births of newborns of at least 34 weeks' gestation hospitalized for early-onset neonatal bacterial infection in France due to 2017 guidelines

	EONI total		Non-severe EONI		Severe EONI		Severe EONI with identified pathogen		EONI with NICU		EONI without NICU		Late-onset NBI total	
Slope change	0.04	p=0.013	0.03	p=0.041	0.01	p=0.001	0.01	p=0.013	0.01	p=0.033	0.04	p=0.018	N/A	N/A

EONI: early-onset neonatal bacterial infection; NICU: neonatal intensive care unit; NBI: neonatal bacterial infection

eTable 7. Mean cost in euro per stay of hospitalization for an early-onset neonatal bacterial infection - France, 2014-2023

	N	Total EONI admission Mean [95% CI]	No NICU admission Mean [95% CI]	NICU admission Mean [95% CI]
2014	12 038	4404 [4219 – 4590]	2411 [2373 – 2449]	10 241 [9750 – 10 911]
2015	11 458	4320 [4120 – 4519]	2264 [2226 – 2303]	10 444 [9711 – 11 177]
2016	9994	4534 [4343 – 4724]	2416 [2370 – 2462]	10 090 [9468 – 10 712]
2017	8589	4824 [4595 – 5053]	2383 [2336 – 2430]	10 982 [10 254 – 11 711]
2018	6280	5265 [4989 – 5541]	2341 [2285 – 2397]	12 069 [11 251 – 12 886]
2019	5075	6047 [5722 – 6372]	2447 [2382 – 2513]	12 327 [11 530 – 13 124]
2020	4310	7350 [6882 – 7818]	2627 [2545 – 2709]	13 793 [12 778 – 14 807]
2021	3943	7967 [7387 – 8347]	2846 [2745 – 2948]	13 909 [12 947 – 14 871]
2022	3351	9034 [8217 – 9549]	3238 [3109 – 3366]	15 166 [14 211 – 16 120]
2023	3069	10 305 [9599 – 11 011]	3197 [3063 – 3332]	18 089 [16 748 – 19 430]

EONI: early-onset neonatal bacterial infection; NICU: neonatal intensive care unit; 95% CI: 95% confidence intervals

© 2025 Paucard L et al. *JAMA Network Open*.

eREFERENCE

1. Zhang F, Wagner AK, Soumerai SB, Ross-Degnan D. Methods for estimating confidence intervals in interrupted time series analyses of health interventions. *J Clin Epidemiol*. 2009;62(2):143-148. doi:10.1016/j.jclinepi.2008.08.007

© 2025 Paucard L et al. *JAMA Network Open*.
