# Supplementary material for: Hospital Admissions After Early-Onset Neonatal Bacterial Infection Management Guidelines in France
Source: JAMA Netw Open. 2025 Nov 26;8(11):e2545436. doi: 10.1001/jamanetworkopen.2025.45436 (PMC12658671; doi:10.1001/jamanetworkopen.2025.45436)
Supplement: Supplement 2. — Data Sharing Statement [file jamanetwopen-e2545436-s002.pdf]

Data Sharing Statement

Paucard. Hospital Admissions After Early-Onset Neonatal Bacterial Infection Management Guidelines in France. *JAMA Netw Open*. Published November 26, 2025. doi:10.1001/jamanetworkopen.2025.45436

Data
Data available: No

Additional Information
Explanation for why data not available: Aggregated monthly data were used for the time series analysis and are available from the corresponding author on reasonable request. The pseudonymized data extracted from the PMSI are only accessible to accredited structures and can be accessed with the permission from the Agence Technique de l′Information sur l′Hospitalisation (ATIH). More information is available on: https://restitutions.atih.sante.fr/#pdh_modalite_acces.
